# PCR-Based Seamless Genome Editing with High Efficiency and Fidelity in *Escherichia coli*

**DOI:** 10.1371/journal.pone.0149762

**Published:** 2016-03-28

**Authors:** Yilan Liu, Maohua Yang, Jinjin Chen, Daojiang Yan, Wanwan Cheng, Yanyan Wang, Anders Thygesen, Ruonan Chen, Jianmin Xing, Qinhong Wang, Yanhe Ma

**Affiliations:** 1 Tianjin Institute of Industrial Biotechnology, Chinese Academy of Sciences, 32 XiQiDao, Tianjin Airport Economic Area, Tianjin, 300308, China; 2 National Key Laboratory of Biochemical Engineering, Institute of Process Engineering, Chinese Academy of Sciences, Beijing, 100190, PR China; 3 Center of Bioprocess Engineering, Department of Chemical and Biochemical Engineering, Technical University of Denmark, DK-2800, Lyngby, Denmark; Imperial College London, UNITED KINGDOM

## Abstract

Efficiency and fidelity are the key obstacles for genome editing toolboxes. In the present study, a PCR-based tandem repeat assisted genome editing (TRAGE) method with high efficiency and fidelity was developed. The design of TRAGE is based on the mechanism of repair of spontaneous double-strand breakage (DSB) via replication fork reactivation. First, *cat-sacB* cassette flanked by tandem repeat sequence was integrated into target site in chromosome assisted by Red enzymes. Then, for the excision of the *cat-sacB* cassette, only subculturing is needed. The developed method was successfully applied for seamlessly deleting, substituting and inserting targeted genes using PCR products. The effects of different manipulations including sucrose addition time, subculture times in LB with sucrose and stages of inoculation on the efficiency were investigated. With our recommended procedure, seamless excision of *cat-sacB* cassette can be realized in 48 h efficiently. We believe that the developed method has great potential for seamless genome editing in *E*. *coli*.

## Introduction

**Fig 1:**
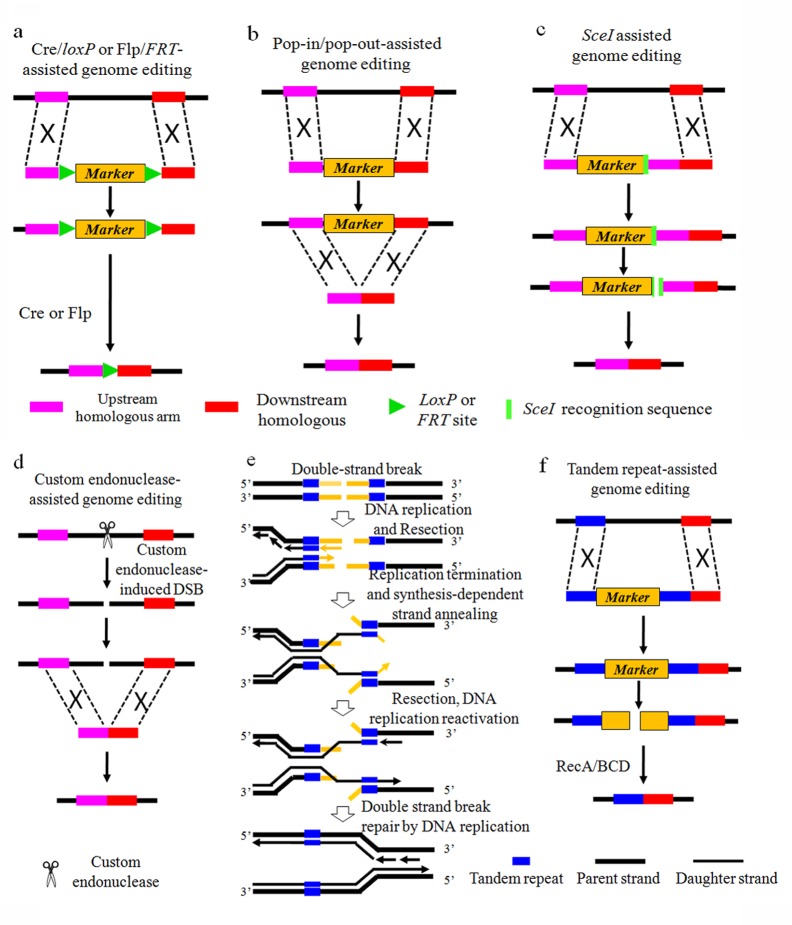
Tandem repeat assisted genome editing (TRAGE) in comparison with the four reported strategies. (a) Cre/*loxP* or Flp/*FRT* systems. (b) The Pop-in/Pop-out strategy. (c) *SceI* assisted method. (d) Custom endonucleases assisted gnome editing method. (e) A model for the current understanding of how RecBCD responds to DSBs in front of the replication fork. (f) The model for TRAGE: First, desired DNA fragment with the selectable marker flanked by tandem repeats was introduced into the target site via intermolecular homologous recombination assisted by Red enzymes. Then, seamless excision of the selectable marker was realized via DSB repair based on intramolecular homologous recombination among the tandem repeats. The excision of selection cassette was realized via replication fork reactivation by the mechanism shown in (e)

Genome editing can introduce predetermined sequence changes to the targeted gene, which could reprogram biological systems for numerous applications. *Escherichia coli* is one of the most important microorganisms for the production of various chemicals such as amino acids, taxol, fatty acids, alkanes, succinate, and so on [1–4]. Therefore, genome editing methods for *E*. *coli* have caught great attention [5,6]. For *E*. *coli*, due to the existence of intracellular exonucleases that degrade linear DNA, linear DNA without protection cannot mediate targeted gene editing like yeast [7]. A simple and efficient way to protect linear DNA with λ Red recombinases was developed to solve this problem [8]. Thereafter, genome editing strategies based on this technology thrived and could be divided into four classes in *E*. *coli*:

The first class is using Cre*/loxP* [9] or Flp*/FRT* [10] systems (Fig 1A), to realize genome editing in *E*. *coli*. However, scar sequence (*FRT* or *loxP*) would be left behind unavoidably. The second class is the "pop-in/pop-out" method (Fig 1B) assisted by double-selection cassettes such as *galK*, *thyA*, *tolC*, *rpsL*, or *tetA-sacB* [11–16]. Seamless genome editing was achieved for the first time. However, the manipulation process for one gene cost nearly a whole week. The third class is assisted by repeat sequence and endonuclease *SceI* (Fig 1C) [17, 18]. This method can facilitate seamless genome editing with high efficiency but high risk of introducing off-target mutations, because *SceI* does not have strict substrate specificity [19]. The fourth class, which has drawn great attention recently, is assisted by sequence-specific endonucleases such as zinc fingers nucleases (ZFNs), transcription activator-like effector nucleases (TALENs), or the clustered regularly interspaced short palindromic repeats (CRISPR)-associated Cas9 endonuclease for genome editing with high efficiency (Fig 1D) [20–27]. Since this strategy is realized by cleaving target sequence, extra mutation has to be introduced into genome to prevent cleaving in the original sequence when gene insertion or single-base substitution was conducted [6].

In this work, we developed a tandem repeat assisted genome editing method (TRAGE) with high efficiency and fidelity in *E*. *coli*. TRAGE was inspired by the mechanism of replication fork reactivation after double strand breaks (DSBs) repair via homologous recombination [28, 29]. During DNA replication, DSBs would lead to replication fork demise. Homologous recombination could reactivate the replication fork, and result in DSB repair. If DSBs appeared in a region flanked by tandem repeats, which is forward repeat sequence to facilitate intramolecular homologous recombination, the endogenous helicase and nuclease could unwind and hydrolyze DSB ends to produce single-stranded DNA containing the tandem repeats [28,30–32]. Then, the intramolecular homologous recombination assisted by tandem repeats annealing will restart the replication. Finally, the break strands could be repaired with the daughter strands as templates. This will result in the precise excision of the region between the tandem repeats (Fig 1E). Hence, TRAGE was designed (Fig 1F).

## Materials and Methods

### Materials

Polymerase chain reaction (PCR) purification kits, gel extraction kits and QIAprep Spin plasmid miniprep kits were purchased from Axygen (Union City, CA, USA). Primers were synthesized by Sangon Biotechnology (Shanghai, China) (Table 1). Fatty alcohol standards were purchased from Dr Ehrenstorfer GmbH (Augsburg, Germany). Constructed strains are listed in Table 2 and additionally used bacterial strains and plasmids are listed in Table A in S1 File.

**Table 1:** Primers used for genome editing and subsequent testing in this study.

Primers	Sequence		Application
P1		**GTGACGGAAGATCACTTCGCAG**	*cat-sacB* fragment construction
P2		**ATCAAAGGGAAAACTGTCCATATGC**	*cat-sacB* fragment construction
KtesAF		**CCGACGGACTTCTTAAGATGATGAACTTCAACAATGTTTTCCGCTGGCAT**ATGACTCATAAAGCAACGGAGATCCTGACAGGTAAAGTTATGCAAAAATCTCCTGGTGTCCCTGTTGATA	*tesA* deletion
KtesAR		GATTTTTGCATAACTTTACCTGTCAGGATCTCCGTTGCTTTATGAGTCATATAGATACATCAGAGCTTTTACGAG	*tesA* deletion
KtesBF		**ATCACGCATTTCTGCCTGTAATTAGCCCGTAATTCAGACCATTGCACCCA**AAAAATAGCCGGAGGTGAAAACCGTCCGGCTGTTTTTTGCAGTGCTTGTTTCCTGGTGTCCCTGTTGATA	*tesB* deletion
KtesBR		AACAAGCACTGCAAAAAACAGCCGGACGGTTTTCACCTCCGGCTATTTTTATAGATACATCAGAGCTTTTACGAG	*tesB* deletion
KfadMF		**CGTAATCTGGCGGTATTAACCCTGTAATTAATTTGCATAGTGGCAATTTT**ACGTTTTGTGGTGCCGGATGCTCAAGCCGCATCCGGCGACACCCGGAATATCCTGGTGTCCCTGTTGATA	*fadM* deletion
KfadmR		TATTCCGGGTGTCGCCGGATGCGGCTTGAGCATCCGGCACCACAAAACGTATAGATACATCAGAGCTTTTACGAG	*fadM* deletion
ASFP1		**CGTAATCTGGCGGTATTAACCCTGTAATTAATTTGCATAGTGGCAATTTT**CGGTTCTGGCAAATATTCTGAAATG	gene substitution
ASFP2		ATTCCGGGTGTCGCCGGATGCGGCTTGAGCATCCGGCACCACAAAACGTAGCGTTCACCGACAAACAACAGATA	gene substitution
ASFP3		ACGTTTTGTGGTGCCGGATGCTCAAGCCGCATCCGGCGACACCCGGAATTCCTGGTGTCCCTGTTGATA	gene substitution
ASFP4		ATTCCGGGTGTCGCCGGATGCGGCTTGAGCATCCGGCACCACAAAACGTATAGATACATCAGAGCTTTTACGAG	gene substitution
Finp1		**TTCTAGAGAATAGGAACTTCGGAATAGGAACTAAGGAGGATATTCATATG**TCAGGCAGCTTTTTTGCGCTGGCGC	gene insertion
Finp2		AAATATTTTTAGTAGCTTAAATGTGATTCAACATCACTGGAGAAAGTCTTATGGCAATACAGCAGGTACATCACG	gene insertion
Finp3		AAGACTTTCTCCAGTGATGTTGAATCACATTTAAGCTACTAAAAATATTTTCCTGGTGTCCCTGTTGATA	gene insertion
Finp4		AAATATTTTTAGTAGCTTAAATGTGATTCAACATCACTGGAGAAAGTCTTATAGATACATCAGAGCTTTTACGAG	gene insertion
TkfadMF		GCACTGCTCATTACCCTGTCCCTG	*fadM* deletion or substitution
TktesAF		TGCCATGTTCACGGTTGAGGG	*tesA* deletion test
TktesBF	TTTACCTTCAGTACGCACCGCTTTC		*tesB* deletion test
TFinF:	GCCGAATATCATGGTGGAAAATGG		gene insertion test
CatR		TGAGCATTCATCAGGCGGGC	fragment integration test
TkfadMR		AGCATCCGGCACCACAAAACG	*fadM* deletion or substitution
TktesAR		GTCAGGATCTCCGTTGCTTTATGAGTCAT	*tesA* deletion test
TktesBR		GACGGTTTTCACCTCCGGCTATTT	*tesB* deletion test
TfinR		CTGGCGATTGCTCCGTCTGC	gene insertion test
KtesAchiF		**CCGACGGACTTCTTAAGATGATGAACTTCAACAATGTTTTCCGCTGGCAT**ATGACTCATAAAGCAACGGAGATCCTGACAGGTAAAGTTATGCAAAAATCgctggtggTCCTGGTGTCCCTGTTGATA	Chi site addtion test
KtesBchiF		**ATCACGCATTTCTGCCTGTAATTAGCCCGTAATTCAGACCATTGCACCCA**AAAAATAGCCGGAGGTGAAAACCGTCCGGCTGTTTTTTGCAGTGCTTGTTgctggtggGTGACGGAAGATCACTTCGCAG	Chi site addtion test
KfadMchiF		**CGTAATCTGGCGGTATTAACCCTGTAATTAATTTGCATAGTGGCAATTTT**ACGTTTTGTGGTGCCGGATGCTCAAGCCGCATCCGGCGACACCCGGAATAgctggtggTCCTGGTGTCCCTGTTGATA	Chi site addtion test

Note: The bold parts of the primers are homologous 5’ end of the target site; the underlined parts of the primers are homologous 3’ end of the target site; the yellow-highlighted parts of the primers are homologous to *cat-sacB* cassette; The framed parts of the primers are homologous to the exogenous fragment; the lowercase nucleotides in the KtesAchiF KtesBchiF and KfadMchiF primers are the Chi site.

**Table 2:** Strain used and constructed in this study.

Strains	Relevant characteristics	Source
MG	*E*. *coli* K-12 MG1655	Lab collection
MGK1	MGK Δ*fadM*:: *fadM* 3’50*-cat-sacB*	This study
MGK1S	MG Δ*fadM*	This study
MGK2	MGK1S Δ*tesB*:: *tesB* 3’50*-cat-sacB*	This study
MGK2S	MGK1S Δ*tesB*	This study
MGK3	MGK2S Δ*tesA*:: *tesA* 3’50*-cat-sacB*	This study
MGK3S	MGK2S Δ*tesA*	This study
MGKFSF	MG Δ*fadM*:: *FAR- fadM* 3’50*- cat-sacB*	This study
MGKFSFS	MG Δ*fadM*:: *FAR*	This study
MGFARIN	MG *ldhA*::*FAR- ldhA* 3’50-*cat-sacB*	This study
MGFARINS	MG *ldhA*::*FAR*	This study
MGKA	MGK Δ*tesA*:: *tesA* 3’50-Chi*-cat-sacB*	This study
MGKAS	MG Δ*tesA*	This study
MGKB	MGK Δ*tesB*:: *tesB* 3’50-Chi*-cat-sacB*	This study
MGKBS	MG Δ*tesB*	This study
MGKF	MGK Δ*fadM*:: *fadM* 3’50*-Chi-cat-sacB*	This study

**Note:** MG is the original strain used for genome editing.

### Cultures

Unless otherwise stated, strains were cultivated in LB medium (10 g/L NaCl, 5 g/L Yeast extract, 10 g/L Tryptone) at 37°C with 220 rpm shaking. For the analysis of fatty alcohol production in engineered strains, colonies of each strain were cultivated in LB medium overnight at 37°C with 220 rpm shaking in three replicates. The next day, 1 mL seed culture was inoculated into 50 mL LB containing 10 g/L glycerol in a 250 mL flask, followed by incubation at 37°C with 220 rpm shaking for 24 h.

### Construction of *cat-sacB* fragment

The *cat-sacB* cassette (Sequence shown in S1 File) was obtained via PCR using pEASY-*cat-sacB* as template, Pfu DNA Polymerase and primers P1 and P2 (Table 1). The PCR product was purified and digested with *DpnI* overnight at 37°C. Then the product was purified and stored at -20°C.

### Construction of fragments for gene deletion, substitution and insertion

For the *tesA*, *tesB* and *fad*M deletion, PCR was conducted with Taq DNA Polymerase, the prepared *cat-sacB* fragment as template and primers KtesAF/KtesAR, KtesBF/KtesBR and KfadMF/KfadmR, respectively. The forward primer consisted of three parts: a 50 base pair (bp) fragment homologous to the 5’ end of the target gene, a 50 bp fragment homologous to the 3’ end of the target gene and a 20 bp fragment homologous to the 5’ end of the *cat-sacB* cassette sequentially. The reverse primer consisted of two parts: a 50 bp fragment homologous to the 3’ end of the target gene and a 25 bp fragment homologous to the 3’ end of the *cat-sacB* cassette sequentially. The structure of amplified fragments are shown in Figure Aa in S1 File. In order to avoid duplex PCR with the same homologous region, amplifications of the fragment for genome editing were conducted with primers homologous to the inner part of the prepared *cat-sacB*.

The fragment for gene substitution or insertion was constructed by fusion PCR, composed of two parts (Figure Ab-c in S1 File). The first part was amplified by PCR with DNA from *Marinobacter aquaeolei* VT8 as template. The forward primer contained a 50 bp fragment homologous to the 5’ end of the target site and a 25 bp fragment homologous to the 5’ end of exogenous fragment (ASFP1 or FinP1). The reverse primer contained a 50 bp fragment homologous to the 3’ end of the target site and a 25 bp fragment homologous to the 3’ end of FAR (ASFP2 or FinP2).

The second part was amplified by PCR with the prepared *cat-sacB* fragment as template. The forward primer contained a 50 bp fragment homologous to the 3’ end of the target site and a 20 bp fragment homologous to the 5’ end of *cat-sacB* (ASFP3 or FinP3). The reverse primer contained a 50 bp fragment homologous to the 3’ end of the target site and a 25 bp fragment homologous to the 3’ end of *cat-sacB* (ASFP4 or FinP4). Fusion PCR was conducted with the forward primers for the first part (ASFP1 or FinP1) and reverse primers for the second part (ASFP4 or FinP4) and the obtained two parts as a template.

### Construction of fragment including Chi site for *tesB* deletion

In order to introduce a Chi site into the fragment for the deletion of *tesA*, *tesB* and *fadM*, a crossover hotspot instigator (Chi) site sequence of GCTGGTGG was introduced to the forward primer. PCR was conducted with the primers KtesAchiF/KtesAR, KtesBchiF/KtesBR and KfadMchiF/KfadMR, respectively. The prepared *cat-sacB* fragment was used as template.

### Deletion of the *cat-sacB* cassette to finish seamless genome editing

For each manipulation, purified clones were cultured in 1 mL of LB medium at 37°C while shaking at 260 rpm in three replicates. After 12 h, 1 mL of the cultures were inoculated into 10 mL LB medium with 10% sucrose in a 50 mL baffled Erlenmeyer flask at 37°C while shaking at 260 rpm. The cultures were streaked on an LB agar plate containing 10% sucrose and cultured overnight at 37°C. Thereafter, 400 clones for each editing were picked and streaked on LB plates with and without chloramphenicol (Cp, 34 mg/L), separately. Then colony PCR was conducted to check the seamless deletion of the *cat-sacB* cassette (Table 1) with the recombinants losing Cp resistance. PCR products were sequenced by ShangHai Majorbio Bio-pharm Technology Co. Ltd (Shanghai, China) to confirm the manipulations.

### Quantification of fatty alcohols

For extraction of fatty alcohols, 5 mL samples of fermentation broth were extracted with 2.5 mL ethyl acetate at 10°C, 260 rpm for 2 min. The mixture was shaken vigorously for a few seconds before placement in a rotary shaker incubator. After extraction, the mixtures were left static for 10 min. The organic layer was transferred to a new centrifuge tube. After centrifugation at 12,000 rpm for 5 min, the clear supernatant was collected and filtered through a 0.45 μm millipore filter and injected into a HPLC system (High performance liquid chromatography) with an RID (Refractive Index Detector) for analysis. The quantification of fatty alcohols was performed with an Agilent 1200 HPLC (Agilent, Co. Ltd. USA) equipped with RID and a SilGreen ODS C18 column (4.6 mm × 250 mm, 5 μm). The mobile phase was methanol: water: acetic acid (90:9.9:0.1, v/v/v). The column temperature was 26°C. The flow rate was 1.0 mL/min.

## Results

### Gene deletion, substitution and insertion with TRAGE

**Fig 2:**
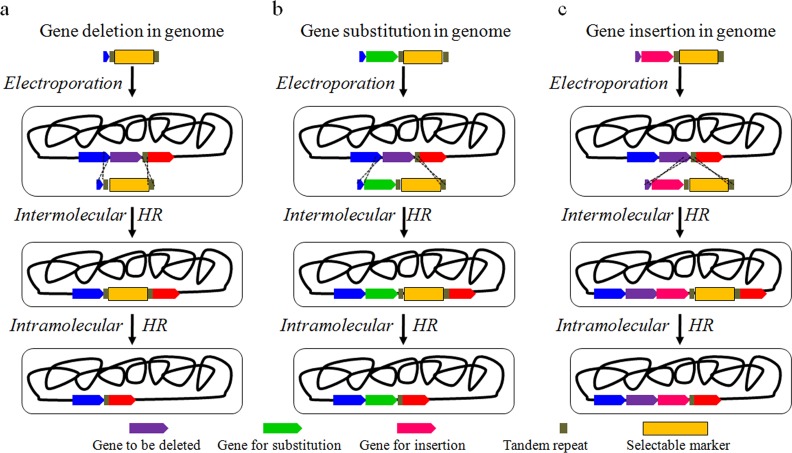
Genome editing strategies with TRAGE in *Escherichia coli*. (a) Gene deletion. (b) Gene substitution. (c) Gene insertion.

**Fig 3:**
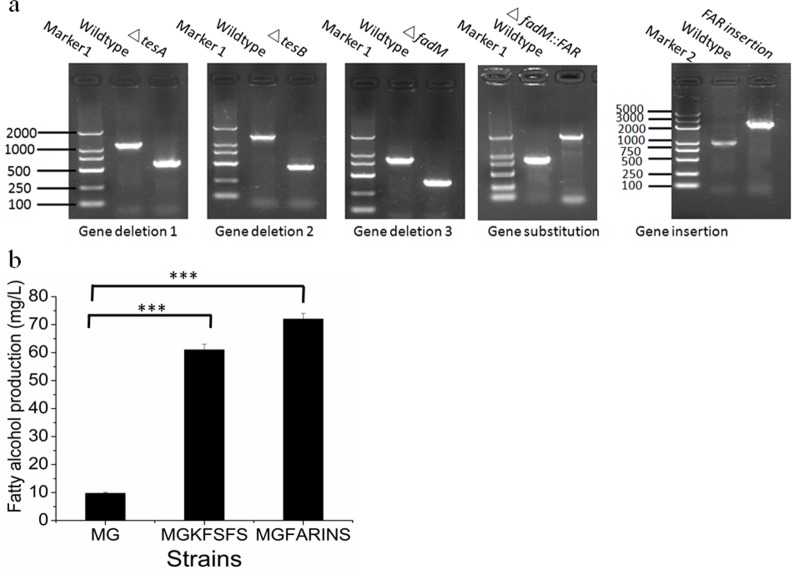
Manipulation results with TRAGE in *E*. *coli*. (a) PCR analysis of seamless deletion of three genes, substitution of one gene and insertion of one gene (b) Fatty alcohol production by strain with integration of *FAR*. Values are the mean of three biological replicates ± standard deviation (n = 3), (_***_) p < 0.001, one-way ANOVA.

Gene deletion was carried out as shown in Fig 2A. First, the constructed DNA fragment with selectable marker flanked by tandem repeats was introduced into the target site via intermolecular homologous recombination assisted by Red enzymes (Protocols A-B in S1 File). Then, seamless excision of selectable marker from the obtained strains was realized via DSB repair based on intramolecular homologous recombination among the tandem repeats. Three unessential genes [33] related to fatty acid metabolism, *fadM*, *tesB* and *tesA* [34,35], were deleted sequentially (Table 2). Fatty acyl-CoA reductase (*FAR*), responsible for fatty alcohol production, was selected for gene substitution and insertion (Fig 2B and 2C). The detailed diagram of the fragments for genome editing is shown in Figure A in S1 File. The electrophoretic results of PCR proved the successful seamless deletion of the three genes *fadM*, *tesA and tesB*, substitution of one gene (Δ*fadM*::*FAR*) and insertion of one gene (*FAR* at the 5’ end upstream of *ldhA*) in *E*. *coli* (Fig 3A). The resulting PCR products were confirmed by sequencing. In addition, HPLC analysis was conducted in MGKFSFS and MGFARINS according to the published method [36]. As shown in Fig 3B, fatty alcohol production was increased from 10 mg/L to 61 and 72 mg/L, respectively. The results proved that the exogenous gene *FAR* was integrated into the genome and expressed in the recombinants successfully.

Additionally, ten other manipulations including deletion of three genes, substitution of two genes and insertion of five functional fragments were realized with TRAGE. In those manipulations, the length of the tandem repeat was ranging from 20 to 50 bp (data not shown). Target modifications were obtained in all manipulations. However, tandem repeats longer than 30 bp are recommended to ensure high efficiency.

### Procedure optimization for TRAGE

**Fig 4:**
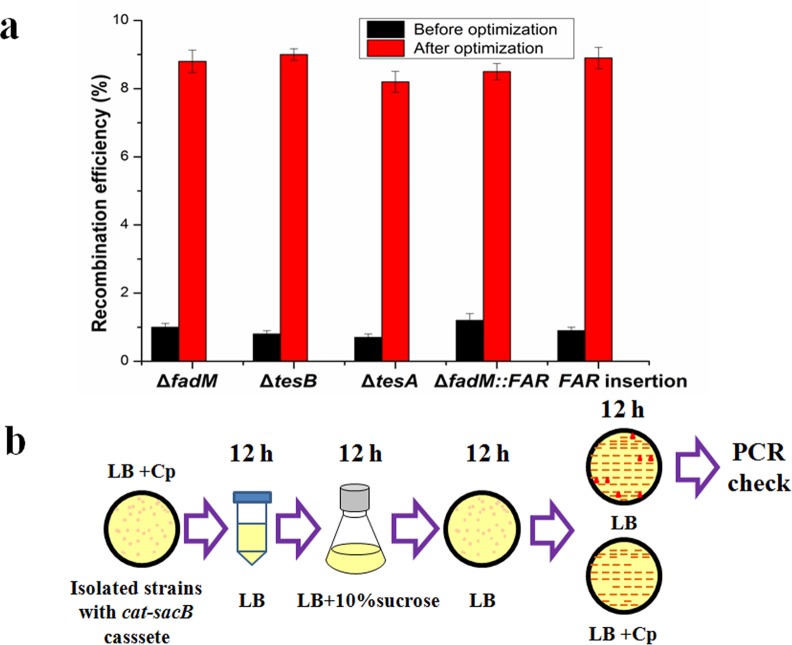
Procedure optimization for TRAGE. (a) Recombination efficiency before and after optimization. The recombination efficiencies were increased from around 1 to 8%. (b) Recommended procedure for the excision of *cat-sacB* by TRAGE with moderate efficiency (about 5%) and short time (around 48 h).

The efficiency of the counter selection step is vital for the application of TRAGE. The effects of different manipulations including sucrose addition time, subculture times in LB with sucrose and stages of inoculation on the efficiency in the counter-selection step were investigated (Table 3). For sucrose addition time, addition of it in the second subculture is preferable for all strains. For example in the seamless deletion of *fadM*, the recombination efficiency was 1.9% when the sucrose was added at the first subculture step. When sucrose was added at the second subculture step, the efficiency increased to 5.3%, which is more than two-fold higher than that in the first subculture. This phenomenon can be attributed to lethal mechanism of *sacB* in *E*. *coli* [37,38]. As to the investigation of effects of subculture times, it is shown that the recombination efficiency increased and reached saturated level as the subculture generation increased to thrice (Table 3). When the subculture generation was increased over thrice, the recombination efficiency decreased without regularity (Table B in S1 File). Maybe that is because strains with *cat-sacB* cassette adapted to medium with sucrose in cultivation process. As to the investigation of the effects of inoculation stage, it is shown that this manipulation is very important and the suitable subculture time is the late stationary phase. Take the seamless deletion of *tesA* for example, the recombination efficiency increased from 0.6% to 8.3% as the inoculation stage changed from log phase to late stationary phase. In summary, the recombination efficiencies for all genome editings were increased from around 1% to 8% under the tested conditions (Fig 4A)

**Table 3:** Effects of different procedures on the recombination efficiency (%).

Genome editing	Time point of sucrose addition			Subculture		Inoculation stage		
	First subculture	Second subculture	Once	Twice	Thrice	Log phase	Stationary phase	Late stationary phase
Δ*fadM*	1.9±0.14	5.3±0.47	4.5±0.31	5.4±0.41	6.0±0.27	1.1±0.09	7.3±0.20	8.9±0.41
Δ*tesB*	2.0±0.09	6.1±0.11	4.2±0.23	5.9±0.13	6.3±0.31	0.9±0.06	8.3±0.32	9.1±0.46
Δ*tesA*	1.4±0.14	5.5±0.41	4.4±0.29	5.6±0.31	5.9±0.32	0.6±0.08	7.2±0.28	8.3±0.36
Δ*fadM*:: *FAR*	2.5±0.17	5.2±0.29	4.1±0.21	5.4±0.19	5.9±0.24	1.1±0.09	7.4±0.27	8.5±0.29
*FAR* insertion	2.1±0.20	5.0±0.32	4.4±0.31	5.7±0.27	6.2±0.35	1.0±0.05	7.4±0.35	9.1±0.43

Note: Recombination efficiency = number of clonies without *cat-sacB*/number of total streaked clonies×100%.For the test of time point of sucrose addition, other parameters were kept the same: subculturing twice after sucrose addition and inoculating at the stationary phase. For the test of subculture times, other parameters were kept the same: adding sucrose at the second subculture and inoculating at the stationary phase. For the test of inoculation stage, other parameters were kept the same: adding sucrose at the second subculture and subculturing thrice after sucrose addition, Values are the mean of three biological replicates ± SD

In order to balance the manipulation time and recombination efficiency, a protocol for TRAGE was recommended as shown in Fig 4B based on the results above. The isolated clones with *cat-sacB* cassette was cultured in 1 mL LB medium in a 2 mL tube overnight to reach the late stationary phase (around 12 h). The next day the 1 mL of cultures were inoculated into 10 mL LB medium with 10% sucrose. After grown to the late stationary phase, the cultures were streaked on a LB agar plate. The grown colonies were then transferred on LB agar with and without Cp simultaneously. Finally, colonies, which lost the Cp resistance, were screened for the excision of *cat-sacB* cassette by PCR. Followed the recommended protocol, the excision of *cat-sacB* cassette can be realized in 48 h with efficiency higher than 5% (Table 4).

**Table 4:** Recombination efficiency with the recommended protocol.

Genome editing	Δ*fadM*	Δ*tesB*	Δ*tesA*	Δ*fadM*:: *FAR*	*FAR* insertion
**Efficiency (%)**	5.2± 0.23	5.9±0.35	5.6±0.19	5.6±0.23	6.0±0.30

Note: Parameters for the test: adding sucrose at the second subculture, inoculating at the late stationary phase, subculturing once after sucrose addition. Values are the mean of three biological replicates ± SD

### Enhancing recombination efficiency by introducing Chi site

According to design principles shown in Fig 1E, the stability of tandem repeat was vital to the efficiency of TRAGE. To enhance the recombination efficiency, a protecting strategy for tandem repeat, was employed. It was demonstrated that Chi sites in the bacterial genome could indirectly inactivate intracellular exonucleases partly and protect the sequence upstream of it. As RecBCD complex encountered with Chi site, the structure of the complex changed and its digesting activity decreased [39, 40]. In this study, a Chi site was introduced to the 3’ end of the upstream tandem repeat sequence to prevent the nascent single-strand tandem repeat sequence from being degraded. Finally, this strategy enhanced the recombination efficiency by 22%, 24%, and 19% for the deletion of *tesA*, *tesB* and *fadM* from the MG strain, respectively.

## Discussion

In this study, spontaneous DSBs coupled with tandem repeat assisted genome editing method was designed. Based on the speculated mechanism (Fig 1F), the amount of spontaneous DSBs in the selectable marker is crucial for the designed method. According to the recent report, the frequency of spontaneous DSBs in *E*. *coli* is about 1% cells per generation [30]. Hence, about 10^7^(10^9^×1%)DSBs will happen in 1 mL culture as it was cultured from OD_600_ of 1 to 2 (∼2×10^9^ cells/mL). Since the genome size of *E*. *coli* is about 4.6×10^6^, the average DSB frequency for each site was about 2.17(10^7^/4.6×10^6^) during this process, on the assumption that the DSBs are evenly distributed. If the specific selectable marker (herein, *cat-sacB* cassette, 2932 bp) flanked by tandem repeat was integrated into the *E*. *coli* genome, there would be more than 6300 (2.17×2932) spontaneous DSBs in the *cat-sacB* cassette in 1 mL culture as it was cultured from OD of 1 to 2. Due to this large number of DSBs, TRAGE should be applicable. The successful gene deletion, substitution and insertion with TRAGE proved this speculation.

Thereafter, the procedure for TRAGE was optimized. Based on the mechanism of TRAGE design (Fig 1F), the *cat-sacB* cassette is deleted during the repair of DSB process via replication fork reactivation. The abundance of DSB is thereby vital for the manipulation efficiency. Since, the number of DSBs is in direct proportion to cell density [30], inoculation at late stages should be better, which was confirmed by the manipulation results (Table 3). The recombination efficiency of inoculation at the late stationary phase was more than seven fold higher than the log phase for each genome editing. Besides, the lethal effect of *sacB* is slow as levansucrase coded by *sacB* produce levan which accumulate in the periplasm of *E*. *coli* and kill the cell [37,38], after a certain period of time. If there is small amount of cells and DSB in the culture when sucrose was added, it is possible to get strains resistant to sucrose not strains losing *cat-sacB* cassette, which will decrease the efficiency of the counter-selection step. This speculation was confirmed by the results that the recombination efficiency with sucrose addition in the second subculture was more than two fold higher than in the first subculture. Finally, according to design principles shown in Fig 1E, the introduction of a Chi site to protect tandem repeat sequence and enhance the recombination efficiency was conducted. The results proved the mechanism speculation, but the effect is not as remarkable as expected. Maybe that is because the introducing of Chi site protected not only the tandem repeat sequence but also itself, while degradation of the Chi site itself is the precondition for recombination. Therefore, the introduction of the Chi site is not recommended for the application of TRAGE.

TRAGE is not only applicable in *E*. *coli*, it also present advantages over the reported methods. Compared with Cre/loxP or Flp/FRT systems, the recombination efficiency is similar. However, TRAGE could facilitate genome editing with no scar sequence left behind. Compared with the "pop-in/pop-out" method, which needs two rounds of electrotransformation assisted by Red enzymes, TRAGE only needs one and thus reduces the manipulation time by 50%. As to recombination efficiency for the marker excision, TRAGE could give higher efficiency. Because, for pop-in/pop-out method, the editing template for marker deletion was introduced to cells via a second electrotransformation, while for TRAGE, it exists in all cells (Fig 1F). Furthermore, it was demonstrated that manipulation with Red enzymes might introduce unwanted recombination events [5]. Unlike the "pop-in/pop-out" method, the marker excision step with TRAGE does not need Red enzymes. Thus, TRAGE provide higher fidelity. Compared with the *SceI* assisted method, TRAGE gives lower recombination efficiency but higher fidelity. Because, the low substrate specificity with *SceI* [19] and too much fragments homologous to the recognition sequence in *E*. *coli* genome (Table C in S1 File), might introduce off-target mutations during the manipulation process. Compared with the sequence-specific endonuclease system such as CRISPR-Cas, with which plasmids for endonucleases must be constructed, the manipulation of TRAGE is much simpler. Besides, for gene insertion or single-base substitution, TRAGE is a better choice. As extra mutation has to be introduced into the target genome to prevent cleaving in the original sequence for endonucleases system [6].

In conclusion, a PCR-based genome editing method with high efficiency and fidelity in *E*. *coli* was developed. Since DSBs and homologous recombination are ubiquitous in most organisms [31,41–43], this method should be applicable to many other organisms or cell types if there is reported double selectable maker for the target organism, such as *spc-mazF* in *Bacillus subtilis* [44]. The limitation of the developed method is the low lethal effect of *sacB*, which affect the manipulation efficiency. As we found that, when strains with and without the *cat-sacB* cassette were cultivated, diluted to same concentration and spread on LB plate with 10% sucrose, colony numbers were similar. Thus, it is possible to enhance the efficiency of TRAGE with an effective counter selectable marker in the future.

## Supporting Information

S1 File (DOC)
