# Supplementary material for: PCR-Based Seamless Genome Editing with High Efficiency and Fidelity in Escherichia coli
Source: PLoS One. 2016 Mar 28;11(3):e0149762. doi: 10.1371/journal.pone.0149762 (PMC4809717; doi:10.1371/journal.pone.0149762)
Supplement: S1 File — (DOC) [file pone.0149762.s001.doc]

Supplementary information

**Seamless genome editing with high efficiency and fidelity in *Escherichia coli***

Yilan Liu^1^, Maohua Yang^2^, Jinjin Chen^2^,, Daojiang Yan^2^, Wanwan Cheng^2^, Yanyan Wang^2^, Anders Thygesen^3^, Ruonan Chen ^2^ , Jianmin Xing^2^, Qinhong Wang^1^, Yanhe Ma^1^

^1^Tianjin Institute of Industrial Biotechnology, Chinese Academy of Sciences, 32 XiQiDao, Tianjin Airport Economic Area, Tianjin 300308, China

^2^National Key Laboratory of Biochemical Engineering, Institute of Process Engineering, Chinese Academy of Sciences, Beijing 100190, PR China;

^3^Center of Bioprocess Engineering, Department of Chemical and Biochemical Engineering, Technical University of Denmark, DK-2800 Lyngby, Denmark

This file includes:

Supplementary Protocols A-B

Supplementary Figures A

Supplementary Tables A-C

*Cat-sacB* Nucleotide sequences

**Supplementary Protocols A. Transform pKD46 to target strain**

1. Grow 1 mL culture of target strain overnight in LB (10 g/L NaCl, 5 g/L Yeast extract, 10 g/L Tryptone)
2. Add 0.5 mL of the overnight culture to 50 mL of LB medium in a 250 mL baffled Erlenmeyer flask. Grow cells with shaking until the cells reach an OD600 of 0.4~0.6.
3. Rapidly chill both cultures in ice-water slurry; Swirl the flasks gently. Leave on ice for 5～10 min. Label and chill two 35 mL centrifuge tubes for cells.
4. Transfer both cultures to the centrifuge tubes at 4,000 g for 15 min at 4 °C (Eppendorf centrifuge 5810R). Pour off supernatant.
5. Add 1 mL of ice-cold sterile distilled H_2_O to each tube and gently suspend cells. Add another 30 mL of ice-cold distilled H_2_O to each tube, seal and gently invert to mix, again without vortexing. Centrifuge tubes at 6,000 g for 15 min at 4 °C.
6. Promptly decant the supernatant very carefully from each tube and gently suspend each cell pellet in 1 mL of ice-cold distilled 10% glycerol to each tube and gently suspend cells. Add another 30 mL of ice-cold distilled 10% glycerol to each tube seal and gently invert to mix, again without vortexing. Centrifuge tubes again as mentioned in Step 5.
7. Promptly decant the supernatant very carefully from each tube and gently suspend cells in 200 μl of sterile ice-cold distilled 10% glycerol keep on ice until used.
8. To introduce pKD46 into the electrocompetent cells (from Step 7) by electroporation, chill the desired number of labeled 0.2-cm electroporation cuvettes on ice. Turn on the MicroPulser and set to “Ec2”, transfer the mixture of cells and plasmid to the cold electroporation cuvette and tap the suspension to the bottom. Place the cuvette right. Pulse once.
9. Remove the cuvette from the chamber and immediately add 1 mL of SOC medium to the cuvette. Transfer cells to a 2 mL tube and incubate at 37 °C for 1 hour, shaking at 220 rpm.
10. Plate cultures on an LB agar plate containing ampicillin 100 mg/L. Next day pick isolated colonies and test for the presence of pKD46.

**Supplementary Protocols B. Integration of designed fragments into genome by intermolecular homologous recombination assisted by Red enzymes.**

1. Grow a 1 mL culture of the target strain contain pKD46 overnight in LB containing Amp 100 mg/l at 30 °C
2. Add 0.5 mL of the overnight culture to 50 mL of LB medium containing Amp 100 mg/l in a 250-ml baffled Erlenmeyer flask at 30 °C shaking at 220 rpm. Grow cells with shaking until the cells reach an OD600 of 0.1. Add L-arabinose 0.75 g and Grow cells with shaking until the cells reach an OD600 of 0.6
3. Make electrocompetent cells
4. Chill the desired number of labeled 0.2-cm electroporation cuvettes on ice. Mix the electrocompetent cells the prepared fragments, then transfer the mixture to chilled electroporation cuvettes, Tap the suspension to the bottom. Turn on the MicroPulser and set to “Ec2”, place the cuvette right. Pulse once.
5. Immediately after electroporation, add 1 mL of LB medium to the cuvette. Transfer cells to a 2 mL tube and incubate at 30 °C for 3 hour, shaking at 140 rpm.
6. Plate cultures on an LB agar plate containing chloramphenicol (34 mg/L). Pick colonies and test for selecting recombinant clones containing fragment from step 1.

**
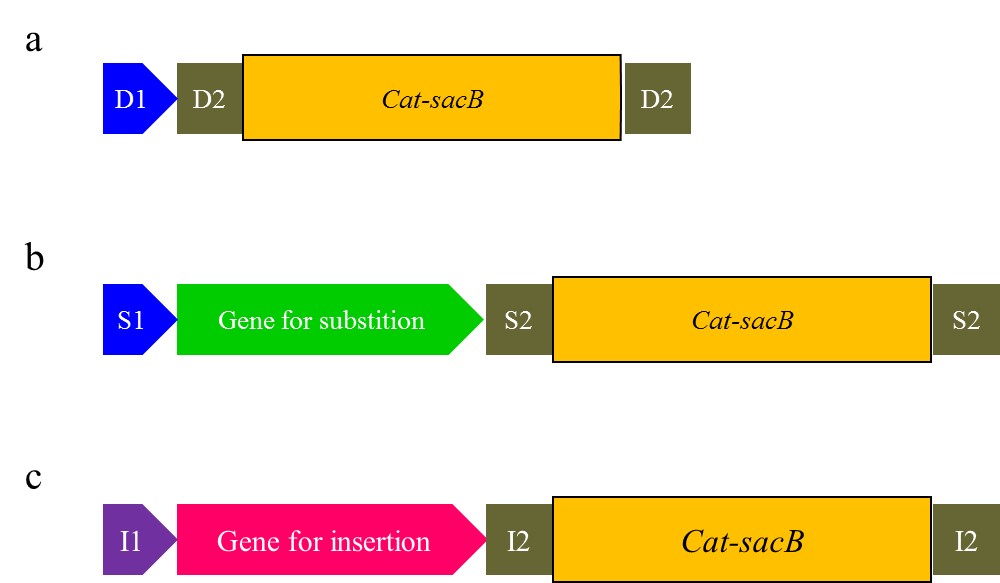
**

**Supplementary Fig A.** Diagram of the fragments for genome editing: gene deletion (a), substitution (b) and insertion (c). D1: 50 bp fragment homologous to the 5’ end of the gene to be deleted; D2: 50 bp fragment homologous to the 3’ end of the gene to be deleted. S1: 50 bp fragment homologous to the 5’ end of the gene to be substituted; S2: 50 bp fragment homologous to the 3’ end of the gene to be substituted. I1: 50bp fragment homologous to the 5’ end of the site insertion; I2: 50 bp fragment homologous to the 3’ end of the site insertion.

**Supplementary Table A Plasmids, strains and primers used in this study**

| **Plasmids/ Strains** | **Relevant characteristic(s)** | | | **Reference/source** |
| --- | --- | --- | --- | --- |
| **Plasmids** | |  | |  |
| pKD46 | | Bla γβexo temperature conditional pSC101 replicon | Datsenko et al. (2000) | |
| pEASY-cat-sacB | | T-easy vector with cat-sacB casstte | Lab collection | |
| **Strains** | |  |  | |
| *Marinobacter aquaeolei* VT8 | | ATCC 700491 / DSM 11845 / VT8 | Lab collection | |

**Supplementary Table B** Effects of subculture times increasing over three times on recombination efficiency (%)

| **Subculture times** | Δ*fadM* | Δ*tesB* | Δ*tesA* | Δ*fadM* :: *FAR* | *FAR* insertion |
| --- | --- | --- | --- | --- | --- |
| **Four** | 5.4± 0.32 | 6.6±0.37 | .5.2±0.21 | 5.7±0,22 | 6.1±0.31 |
| **Five** | 5.2± 0.34 | 4.9± 0.35 | 6.1± 0.29 | 4.6±0.28 | 5.5±0.16 |
| **Six** | 5.5± 0.41 | 5.7± 0.33 | 4.3± 0.45 | 5.1±0.17 | 5.7±0.33 |

Note: Recombination efficiency=number of colonies without *cat-sacB*/number of total colonies ×100%. For this test, sucrose was adding sucrose at the second subculture; inoculation was performed at the stationary phase. Values are the mean of three biological replicates ± SD

**Supplementary Table C. Fragments in *E. coli* genome homologous to the recognition site of *Sce I***

| **Homologous length** | **Identities** | **Gaps** | **Fragment number** |
| --- | --- | --- | --- |
| 12bp | 12/12(100%) | 0/12(0%) | 3 |
| 11bp | 11/11(100%) | 0/11(0%) | 16 |
| 10bp | 10/10(100%) | 0/10(0%) | 36 |
| 9bp | 9/9(100%) | 0/9(0%) | 230 |

**Nucleotide sequences**

- ***cat-sacB***

GTGACGGAAGATCACTTCGCAGAATAAATAAATCCTGGTGTCCCTGTTGATACCGGGAAGCCCTGGGCCAACTTTTGGCGAAAATGAGACGTTGATCGGCACGTAAGAGGTTCCAACTTTCACCATAATGAAATAAGATCACTACCGGGCGTATTTTTTGAGTTATCGAGATTTTCAGGAGCTAAGGAAGCTAAAATGGAGAAAAAAATCACTGGATATACCACCGTTGATATATCCCAATGGCATCGTAAAGAACATTTTGAGGCATTTCAGTCAGTTGCTCAATGTACCTATAACCAGACCGTTCAGCTGGATATTACGGCCTTTTTAAAGACCGTAAAGAAAAATAAGCACAAGTTTTATCCGGCCTTTATTCACATTCTTGCCCGCCTGATGAATGCTCATCCGGAATTCCGTATGGCAATGAAAGACGGTGAGCTGGTGATATGGGATAGTGTTCACCCTTGTTACACCGTTTTCCATGAGCAAACTGAAACGTTTTCATCGCTCTGGAGTGAATACCACGACGATTTCCGGCAGTTTCTACACATATATTCGCAAGATGTGGCGTGTTACGGTGAAAACCTGGCCTATTTCCCTAAAGGGTTTATTGAGAATATGTTTTTCGTCTCAGCCAATCCCTGGGTGAGTTTCACCAGTTTTGATTTAAACGTGGCCAATATGGACAACTTCTTCGCCCCCGTTTTCACCATGGGCAAATATTATACGCAAGGCGACAAGGTGCTGATGCCGCTGGCGATTCAGGTTCATCATGCCGTTTGTGATGGCTTCCATGTCGGCAGAATGCTTAATGAATTACAACAGTACTGCGATGAGTGGCAGGGCGGGGCGTAATTTTTTTAAGGCAGTTATTGGTGCCCTTAAACGCCTGGTGCTACGCCTGAATAAGTGATAATAAGCGGATGAATGGCAGAAATTCGAAAGCAAATTCGACCCGGTCGTCGGTTCAGGGCAGGGTCGTTAAATAGCCGCTAGATCTAAGTAAATCGCGCGGGTTTGTTACTGATAAAGCAGGCAAGACCTAAAATGTGTAAAGGGCAAAGTGTATACTTTGGCGTCACCCCTTACATATTTTAGGTCTTTTTTTATTGTGCGTAACTAACTTGCCATCTTCAAACAGGAGGGCTGGAAGAAGCAGACCGCTAACACAGTACATAAAAAAGGAGACATGAACGATGAACATCAAAAAGTTTGCAAAACAAGCAACAGTATTAACCTTTACTACCGCACTGCTGGCAGGAGGCGCAACTCAAGCGTTTGCGAAAGAAACGAACCAAAAGCCATATAAGGAAACATACGGCATTTCCCATATTACACGCCATGATATGCTGCAAATCCCTGAACAGCAAAAAAATGAAAAATATCAAGTTCCTGAATTCGATTCGTCCACAATTAAAAATATCTCTTCTGCAAAAGGCCTGGACGTTTGGGACAGCTGGCCATTACAAAACGCTGACGGCACTGTCGCAAACTATCACGGCTACCACATCGTCTTTGCATTAGCCGGAGATCCTAAAAATGCGGATGACACATCGATTTACATGTTCTATCAAAAAGTCGGCGAAACTTCTATTGACAGCTGGAAAAACGCTGGCCGCGTCTTTAAAGACAGCGACAAATTCGATGCAAATGATTCTATCCTAAAAGACCAAACACAAGAATGGTCAGGTTCAGCCACATTTACATCTGACGGAAAAATCCGTTTATTCTACACTGATTTCTCCGGTAAACATTACGGCAAACAAACACTGACAACTGCACAAGTTAACGTATCAGCATCAGACAGCTCTTTGAACATCAACGGTGTAGAGGATTATAAATCAATCTTTGACGGTGACGGAAAAACGTATCAAAATGTACAGCAGTTCATCGATGAAGGCAACTACAGCTCAGGCGACAACCATACGCTGAGAGATCCTCACTACGTAGAAGATAAAGGCCACAAATACTTAGTATTTGAAGCAAACACTGGAACTGAAGATGGCTACCAAGGCGAAGAATCTTTATTTAACAAAGCATACTATGGCAAAAGCACATCATTCTTCCGTCAAGAAAGTCAAAAACTTCTGCAAAGCGATAAAAAACGCACGGCTGAGTTAGCAAACGGCGCTCTCGGTATGATTGAGCTAAACGATGATTACACACTGAAAAAAGTGATGAAACCGCTGATTGCATCTAACACAGTAACAGATGAAATTGAACGCGCGAACGTCTTTAAAATGAACGGCAAATGGTACCTGTTCACTGACTCCCGCGGATCAAAAATGACGATTGACGGCATTACGTCTAACGATATTTACATGCTTGGTTATGTTTCTAATTCTTTAACTGGCCCATACAAGCCGCTGAACAAAACTGGCCTTGTGTTAAAAATGGATCTTGATCCTAACGATGTAACCTTTACTTACTCACACTTCGCTGTACCTCAAGCGAAAGGAAACAATGTCGTGATTACAAGCTATATGACAAACAGAGGATTCTACGCAGACAAACAATCAACGTTTGCGCCAAGCTTCCTGCTGAACATCAAAGGCAAGAAAACATCTGTTGTCAAAGACAGCATCCTTGAACAAGGACAATTAACAGTTAACAAATAAAAACGCAAAAGAAAATGCCGATATTGACTACCGGAAGCAGTGTGACCGTGTGCTTCTCAAATGCCTGATTCAGGCTGTCTATGTGTGACTGTTGAGCTGTAACAAGTTGTCTCAGGTGTTCAATTTCATGTTCTAGTTGCTTTGTTTTACTGGTTTCACCTGTTCTATTAGGTGTTACATGCTGTTCATCTGTTACATTGTCGATCTGTTCATGGTGAACAGCTTTAAATGCACCAAAAACTCGTAAAAGCTCTGATGTATCTATCTTTTTTACACCGTTTTCATCTGTGCATATGGACAGTTTTCCCTTTGAT
